# *ATM*, *BLM*, and *CDH1* gene co-mutations in a high-grade endometrial stromal sarcoma patient with multiple abdominal cavity metastases: a case report and literature review

**DOI:** 10.1186/s12877-024-05201-z

**Published:** 2024-07-15

**Authors:** Nan Li, Yaxin Yan, Yaxing Li, Yanyan Yang, Congwei Dai, Na Li

**Affiliations:** 1 Department of Oncology, Hebei General Hospital, Shijiazhuang, 050051 Hebei China; 2 Teaching and Research Section of Oncology, Hebei Medical University, Shijiazhuang, 050011 Hebei China; 3 Department of Obstetrics and Gynecology, Hebei General Hospital, Shijiazhuang, 050051 Hebei China

**Keywords:** High-grade endometrial stromal sarcoma, PARPi, Next generation sequencing, *ATM*, *BLM*, *CDH1*

## Abstract

**Background:**

High-grade endometrial stromal sarcoma (HG-ESS) is a rare malignant tumor with poor prognosis. To overcome the limitations of current treatment for advanced patients, the intervention of targeted drug therapy is urgently needed.

**Case presentation:**

A 74-year-old married woman who presented with abdominal distension and lower abdominal pain was admitted to Hebei General Hospital. After surgery, immunohistochemical staining revealed a malignant tumor which was consistent with HG-ESS. Tumor recurrence occurred 2 months after surgery. Then the patient underwent chemotherapy with two courses but responded poorly. Subsequently we observed *ATM*, *BLM*, and *CDH1* co-mutations by Next Generation Sequencing (NGS). Then the patient received pamiparib, which resulted in a 10-month progression-free survival (PFS) and is now stable with the administration of sintilimab in combination with pamiparib and anlotinib.

**Conclusions:**

Due to the successful use of poly ADP-ribose polymerase inhibitor (PARPi) on HG-ESS, we suggest that the selection of effective targeted drugs combined with anti- programmed death-1 (PD-1) drug therapy based on genetic testing may become a new option for the treatment of homologous repair deficient (HR-deficient) HG-ESS.

## Background

Endometrial stromal sarcoma (ESS), derived from endometrial stromal cells, accounts for approximately 1% of all uterine malignancies. According to the latest 2020 WHO classification, ESSs can be divided into four categories: endometrial stromal nodule (ESN), low-grade endometrial stromal sarcoma (LG-ESS), high-grade endometrial stromal sarcoma (HG-ESS) and undifferentiated uterine sarcoma (UUS) [1]. HG-ESS, a highly rare event with a high rate of focal and metastatic recurrence and poor prognosis, is of unique biological behaviors. The treatment for early (stage I to stage II) HG-ESS patients is mainly surgery, including total hysterectomy plus bilateral adnexectomy. However, due to its uncertainty in improving patient prognosis, whether tumor cell reduction should be performed for advanced (stage III to IV) HG-ESS patients is unclear. Moreover, radiotherapy or chemotherapy, the most common postoperative adjuvant therapy strategy, is not effective for treating HG-ESS. In this study, we report a case of HG-ESS mutated with the *ATM*, *BLM*, and *CDH1* genes, which achieved satisfactory clinical results with the oral targeted drug pamiparib.

## Case presentation

**Fig. 1:**
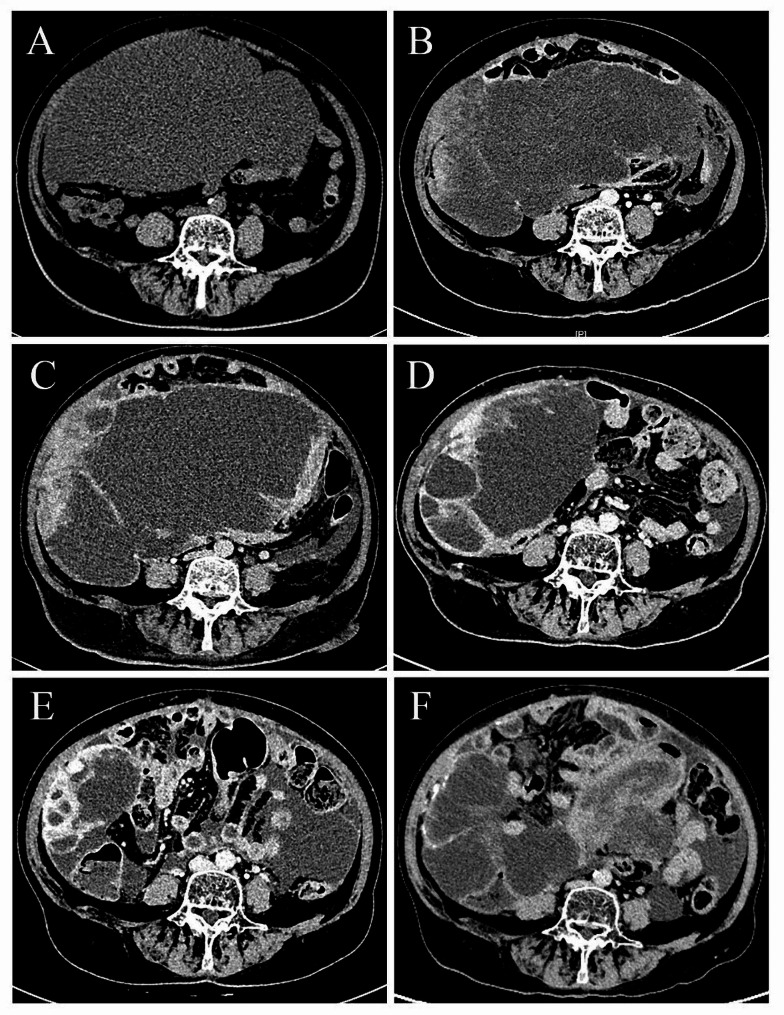
Radiologic features at different time points. **A** Preoperative CT scan (unenhanced CT) (2022-4). **B** Tumor recurrence after surgery (enhanced CT) (2022-6). **C** After 2 cycles of chemotherapy, no significant relief was revealed using enhanced CT (2022-8). **D** After 6 months of pamiparib treatment, an enhanced CT scan indicated PR (2023-2). **E** A 10-month PFS was achieved using pamiparib (enhanced CT) (2023-6). **F** The latest enhanced CT review (2023-9)

A 74-year-old married woman who presented with abdominal distension and lower abdominal pain in April 2022 was admitted to Hebei General Hospital. Pelvic abdominal computed tomography (CT) suggested that there was a mixed-density space-occupying lesion at the base of the uterus as well as abdominal pelvic effusion (Fig. 1A). Gynecological ultrasound indicated that there was a mixed echo mass in the uterine (129 × 124 × 105 mm mixed echo mass, the inner part of which was heterogeneous high echo with strong echo spots) and a pelvic-abdominal cavity mixed echo mass (233 × 208 × 131 mm mixed echo mass above the uterus) with an abdominal fluid dark area. Serological examination revealed a high level of CA125 (162.300U/ml). The patient underwent exploratory laparotomy under general anesthesia on 2022-04-20.

**Fig. 2:**
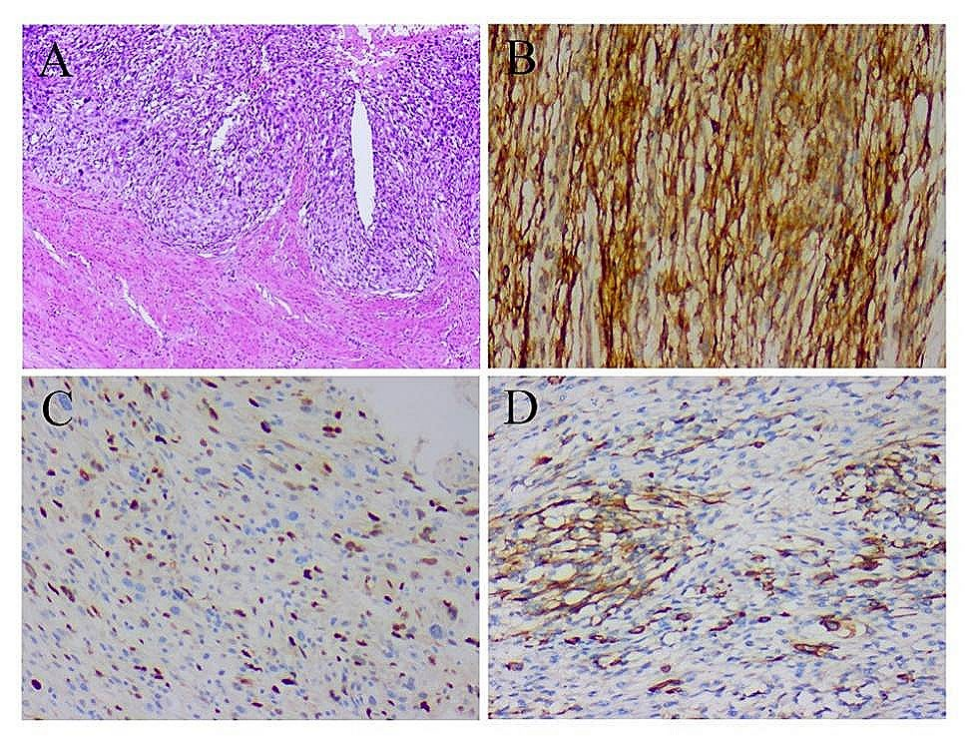
Histopathologic features of HG-ESS. **A** Hematoxylin and eosin (HE) staining (40×) revealed striated muscle differentiation in HG-ESS. **B** immunohistochemical (IHC) examinations (100×) of HG-ESS tissue was positive for CD10. **C** IHC examinations for CyclinD1. **D** IHC examinations for MyoD1

The laparotomy findings were an irregular cystic solid mass with a diameter of approximately 30 cm in the pelvic-abdominal cavity adhering to the posterior wall of the uterus, intestinal tubes, and peritoneum, and the surface of the greater omentum was covered with focal nodules. Intraoperative frozen sections revealed spindle malignancy cells. Subsequently, total abdominal hysterectomy, bilateral adnexectomy, partial greater omentectomy, pelvic abdominal mass resection, and pelvic adhesion release were performed. In histopathological analysis, the resected specimen exhibited diffusely distributed tumor cells in high-magnification images. Tumor cells rendered short spindle shapes and scattered multinucleated tumor giant cells could be seen. Nuclear chromatin was fine and uniform, and the mitotic index was > 10/10 in high-power fields (Fig. 2A). Immunohistochemistry showed positive staining for cell CyclinD1, CD10, Caldesmon, and Vimentin in the tumor cells, with some tumor cells also demonstrating positive labeling for MyoD1, CKpan, and Myogenin (Fig. 2B, C, D). Tumor cells were negative for estrogen receptor, smooth muscle actin, CD117, and S100. The Ki-67 labeling index was approximately 40%. Based on immunohistochemical staining, it was consistent with HG-ESS of the endocervix with cartilage, bone, and striated muscle differentiation.

The patient refused further radiotherapy and chemotherapy. Unfortunately, tumor recurrence occurred 2 months after surgery (Fig. 1B). Thus, the patient was given injections of liposomal doxorubicin and ifosfamide for 2 cycles. The growth of solid component enhancement in an outer rim of the tumor in the right flank and septal enhancement were revealed using enhanced CT (Fig. 1C). The patient exhibited ongoing abdominal distention with a progressive increase in abdominal circumference, and the reaction of the cancer to chemotherapy was unsatisfactory. Moreover, the patient had poor tolerance to chemotherapy. Subsequently, hybridization capture-based targeted Next Generation Sequencing (NGS) was performed on the illumina MiSeq platform (Life Healthcare Group Limited, Beijing, China). The patient underwent testing with a 176-gene panel associated with molecularly targeted drugs, immunotherapy, and chemotherapeutic agents for solid tumors. The proportion of quality score of the sequencing data above Q30 in the samples was more than 92% and passed the quality control. It was found that *ATM*, *BLM*, and *CDH1* genes were mutated. Specifically, a substitution of c. 5908 C > T (p.Gln1970*) was identified in exon 39 of the *ATM* gene with a mutation abundance of 1.17%. Additionally, a mutation of c.1937G > T (p.Ser646Ile) was detected in exon 8 of the *BLM* gene with a mutation abundance of 32.05%. The third significant genetic change was a c.2024 A > G mutation in exon13 of the *CDH1* gene (p.Lys675Arg) with 45.72% mutation abundance. The results showed that the tumor belonged to microsatellite stable (MSS) phenotype. Subsequently, the patient received pamiparib 80 mg a day as well as an intraperitoneal infusion of recombinant human endostatin (45 mg d1,60 mg d4,60 mg d8) combined with cisplatin (40 mg d2, d5, d8), respectively. Efficacy was evaluated for partial response (PR) (Fig. 1D). Subsequent single-agent administration of pamiparib 40 mg 2/day maintenance therapy resulted in a 10-month progression-free survival (PFS) (Fig. 1E). Unfortunately, tumor progression reappeared after 10 months and is now stable with the administration of sintilimab in combination with pamiparib and anlotinib (Fig. 1F) (Last follow-up 2023-12-29).

## Discussion

The clinical presentation of HG-ESS is often nonspecific, possessing a high degree of malignancy and aggressiveness associated with a poor prognosis. Histopathological examination remains the definitive standard for diagnosis. Risk factors that have been found to influence overall survival in HG-ESS include disease stage, tumor size, minimum and average CA125 levels, menopausal status, history of uterine smooth muscle tumors, and endometriosis [2]. A study showed that for patients with stage III, the 1-year disease-specific survival (DSS) rate was 26.7%, and 0% for those with stage IV [3]. Analysis of the data revealed that the median survival time (95% CI) for HG-ESS is only 19.9 (17.1–22.1) months, and for each additional 1 cm in tumor size, the survival rate decreases by 2%[4].

The current treatment for HG-ESS predominantly involves surgical intervention complemented by adjuvant systemic therapy and local radiotherapy. However, the efficacy of lymphadenectomy for HG-ESS is controversial [5]. Current studies suggest that a multimodal approach combining surgery, radiotherapy, and chemotherapy may enhance PFS exclusively in patients with early-stage disease [6]. For patients with advanced recurrent metastases, there is currently no optimal standard treatment, and the National Comprehensive Cancer Network guidelines recommend that patients with NTRK-like family member 4 (*SLITRK4*) gene fusions choose targeted therapies such as larotrectinib or entrectinib.

The combination of CDK4/6 inhibitors and aromatase inhibitors has recently been studied as an option for treating ER-positive patients with BCOR-related metastatic HG-ESS [7].

In this case, a 74-year-old woman was diagnosed with *ATM*, *BLM*, and *CDH1* co-mutations in HG-ESS with a lesion characterized by a large mass (30 cm) and late-stage disease, and she did not receive adjuvant therapy after surgery. The patient, whose tumor recurred two months post-surgery, and the response to chemotherapy was unsatisfactory, achieved remission following treatment with the poly ADP-ribose polymerase inhibitor (PARPi) pamiparib.

Advancements in molecular analysis techniques have led to the detection of mutations in various genes associated with HG-ESS, including *YWHAE*,* NUTM2*,* EPC1*,* SUZ12*,* BCOR*,* PHF1*,* ZC3H7B*,* EML4*,* COL1A1*,* PDGFB*,* STRN*,* MDM2*,* CDK4*,* SLITRK4*, etc. [8–17]. With the development of NGS technologies, other mutated genes may also be identified, and could serve as potential therapeutic targets.

In this report, NGS confirmed mutations within the *ATM*, *BLM*, and *CDH1* genes, among which the *ATM* and *BLM* genes were classified as homologous recombination repair (HRR) genes.

The ataxia-telangiectasia mutated (ATM) protein is the most critical initiator of the DNA damage response (DDR) [18], and its signaling pathway involves hundreds of downstream targets that regulate DDR, proliferation, metabolism, and other physiological activities of cells [19, 20]. According to previous reports, the ATM gene is associated with an increased risk of various cancers, such as breast cancer, lung cancer, pancreatic cancer, and melanoma [21–24]. Studies have shown that mutations in the ATM gene can induce sensitivity to PARPi [25–28].

Bloom syndrome protein (BLM), a member of the RecQ family of helicases using the energy from ATP hydrolysis to unwind duplex DNA, plays a crucial role in correcting mismatched bases to reduce DNA damage induced by itself or the external environment [29]. Recently, the relationship between BLM and tumor development has been discovered [30, 31]. For instance, as a potential biomarker for prostate cancer, BLM has attracted the attention of many investigators. Several reports have suggested that *BLM* mutations in prostate cancer increase the sensitivity of patients to PARPi_olaparib [32].

E-cadherin gene (*CDH1*) mutations are considered to be noteworthy contributors to tumor migration and invasion [33]. Studies have elucidated that the reduction of CDH1 expression increases the cytotoxic effect of PARPi on triple-negative breast cancer cells with or without *BRCA* defects by inducing DNA damage, checkpoint activation, cell cycle arrest, and cell apoptosis [34].

Few studies have investigated *ATM*, *BLM*, and *CDH1* gene mutations in high-grade endometrial stromal sarcoma. In the present study, *ATM*, *BLM*, and *CDH1* mutations were detected by NGS. The patient who received PARPi obtained a 10-month-PFS after 2 cycles of peritoneal perfusion of cisplatin and anti-vascular drugs. PARPi combined with anlotinib was applied after subsequent progression.

Alterations in DDR genes have been linked to genomic instability and increased tumor mutational burden, potentially enhancing tumor immunogenicity [35]. The STING signaling pathway is activated with incompletely repaired DNA damage accumulation, thereby enhancing the immune response [36].

Treatment with PARPi may further increase the level of DNA damage and promote the release of neoantigens and the expression of tumor programmed death-ligand 1 (PD-L1) [37]. The potential synergistic effect between PARPi and PD-1/PD-L1 inhibitors has been confirmed in preclinical studies across various tumor types [38]. Accordingly, we managed to control the disease by administering immunotherapy in combination with PARPi and anlotinib following the patient’s disease re-progression.

## Conclusion

Due to the low prevalence of HG-ESS, there is a lack of consensus on diagnostic and treatment strategies, and there is currently no standard treatment for advanced patients with recurrent metastases. Thus, we anticipate that treatment based on the identification of genetic mutations that can be targeted could lead to the exploration of novel therapeutic approaches for HG-ESS. We identified a case of postoperative HG-ESS recurrence in a patient with mutations in the *ATM*, *BLM*, and *CDH1* genes. This patient responded poorly to chemotherapy. PFS resulting from the PARPi (pamiparib) was nearly 10 months in the first stage. After progression, the PARPi was used in combination with multi-targeted tyrosine kinase inhibitor (anlotinib) and anti-PD-1 (sintilimab)to achieve a PR. To our knowledge, this is the first case report of HG-ESS with *ATM*, *BLM*, and *CDH1* mutations successfully treated with PARPi based on genetic alteration information generated by NGS, suggesting that the selection of effective targeted drugs combined with anti-PD-1 drug therapy on the basis of genetic testing may become a new option for the treatment of homologous repair (HR-deficient) HG-ESS.

## Data Availability

No datasets were generated or analysed during the current study.

## Abbreviations

HG-ESS: High-grade endometrial stromal sarcoma
NGS: Next Generation Sequencing
PFS: progression-free survival
PARPi: Poly ADP-ribose polymerase inhibitor
HR: Homologous recombination
ESS: Endometrial stromal sarcoma
ESN: endometrial stromal nodule
LG-ESS: low-grade endometrial stromal sarcoma
UUS: Undifferentiated uterine sarcoma
CT: Computed tomography
PR: Partial response
DSS: Disease specific survival
HE: Hematoxylin and eosin
IHC: immunohistochemical
HRR: Homologous recombination repair
DDR: DNA damage response
ATM: Ataxia-telangiectasia mutated
BLM: Bloom syndrome protein
CDH1: E-cadherin gene
PD-1: Programmed death-1
PD-L1: Programmed death-ligand 1
